# Supplementary figures and images for: BiP/GRP78 is a pro-viral factor for diverse dsDNA viruses that promotes the survival and proliferation of cells upon KSHV infection
Source: PLoS Pathog. 2024 Oct 29;20(10):e1012660. doi: 10.1371/journal.ppat.1012660 (PMC11548844; doi:10.1371/journal.ppat.1012660)

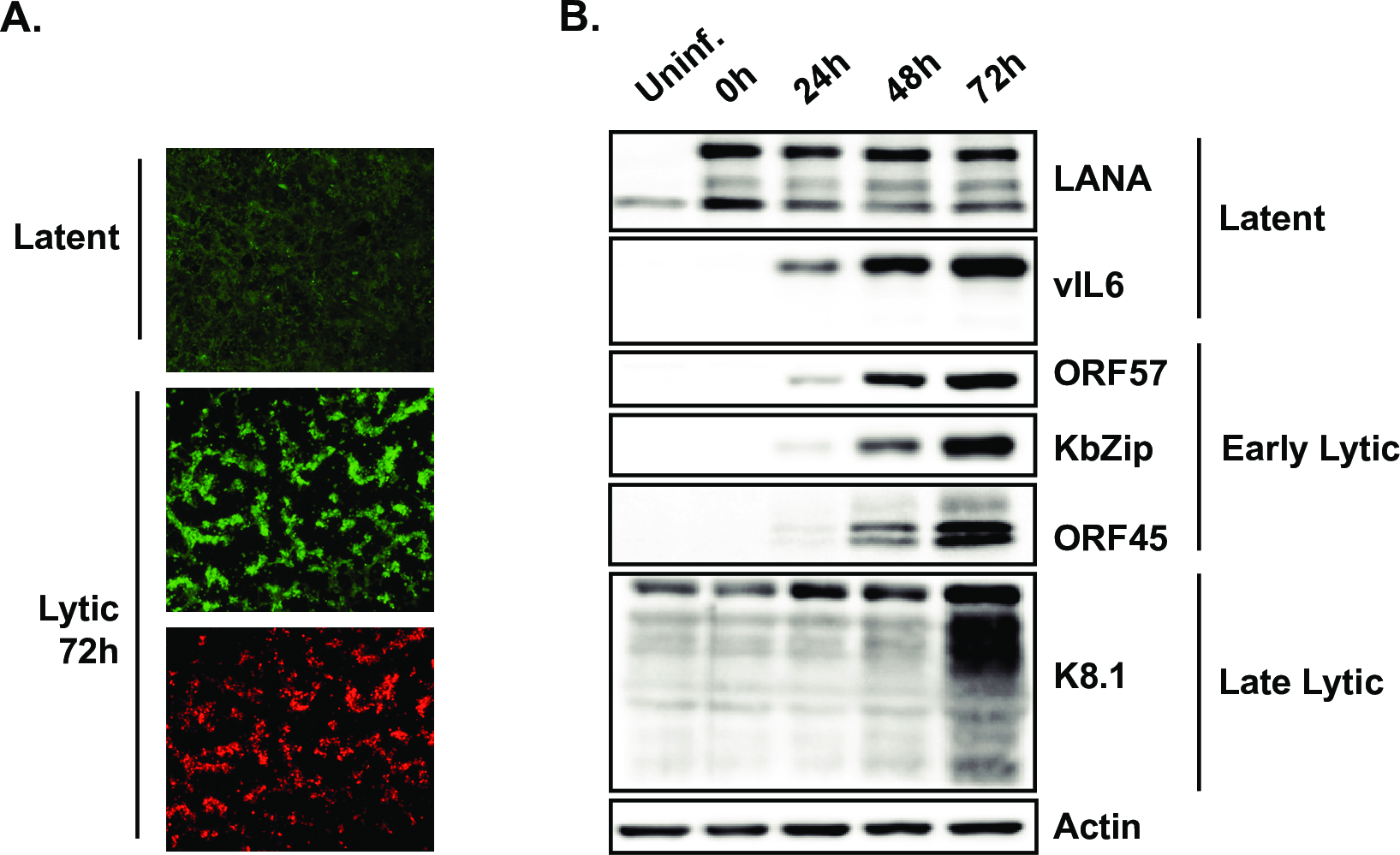

Supplement: S1 Fig — Latently infected iSLK.219 cells were induced to enter the lytic cycle by exogenous expression of RTA following Dox (1 μg/ml) treatment. (A) Imaging of cells at 72h post reactivation showing the expression of the lytic PAN-RFP marker in the population. (B) Immunoblot for viral proteins in iSLK.219 lysates collected at the indicated time points. Images are representative of 3 independent biological replicates. Actin: loading control. (TIF) [file ppat.1012660.s001.tif]

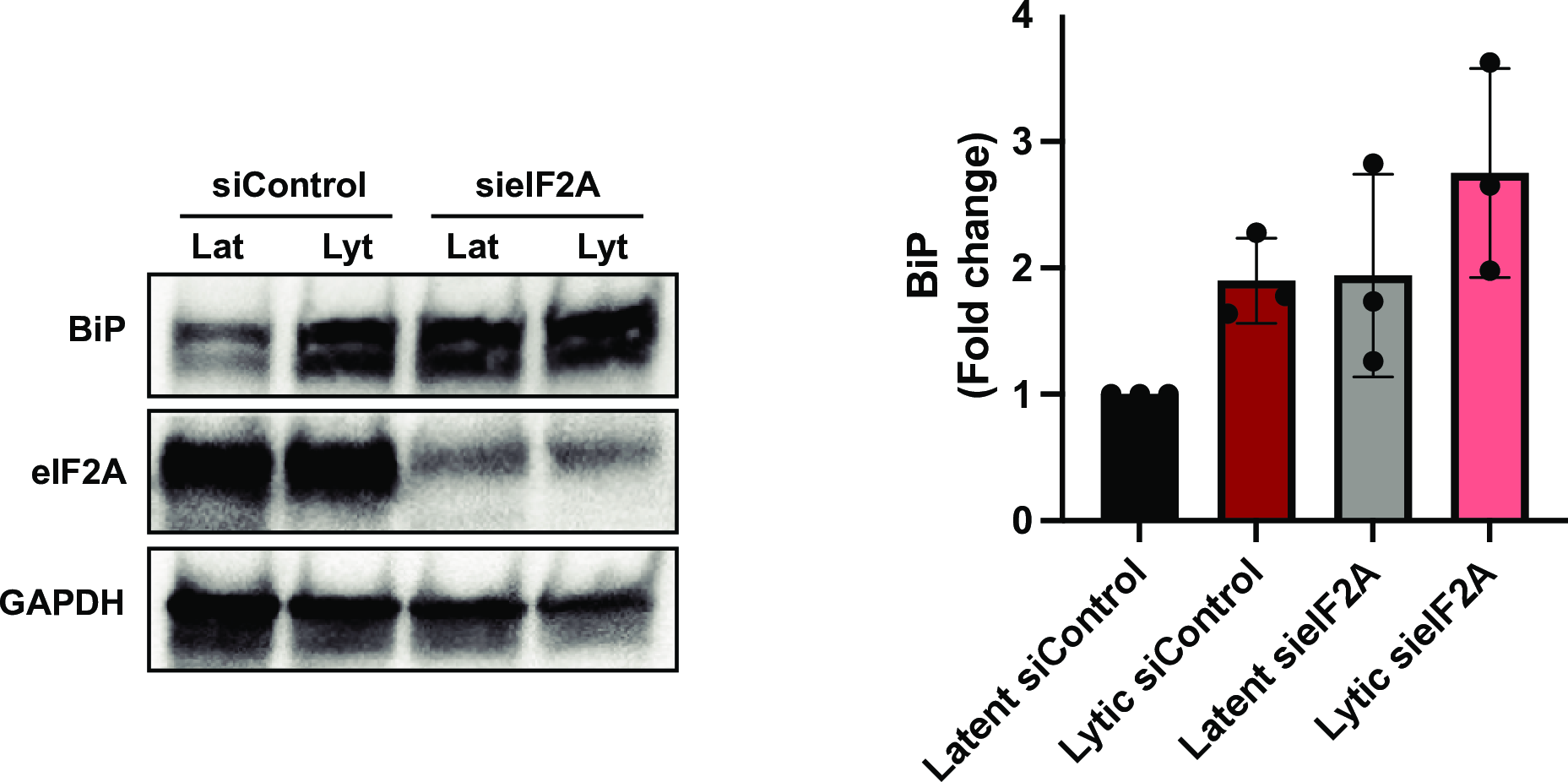

Supplement: S2 Fig — iSLK.219 cells were transfected with a non-targeting siRNA, or an siRNA targeting eIF2A (scbt Cat78713). Cells were grown for 3 days prior to treatment with 1ug/ml dox for 48h. (Left) Whole-cell lysates collected at 48h post-dox treatment were analyzed by immunoblot. GAPDH: loading control. (Right) Image densitometry quantification of the BiP immunoblot. N = 3 (eIF2A antibody proteintech 11233-1-AP, BiP antibody C50B12, GAPDH 14C10). (TIF) [file ppat.1012660.s002.tif]

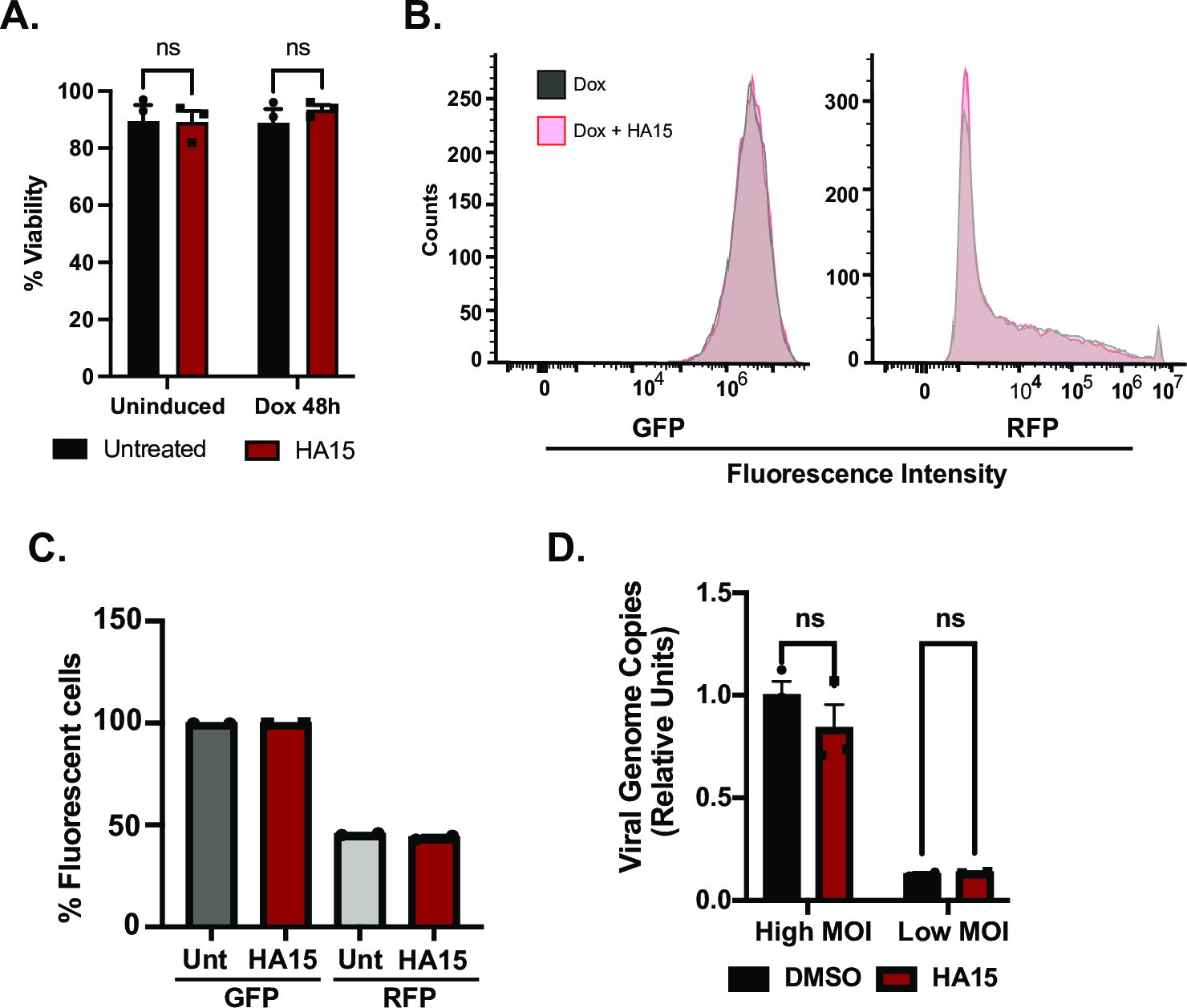

Supplement: S3 Fig — Latently infected iSLK.219 cells were induced to enter the lytic cycle by exogenous expression of RTA following Dox (1 μg/ml) treatment for 24h-48h in the presence or absence of HA15 10 μM. (A) Cells were collected at 48h, stained with trypan blue, and counted to measure viability. (B) Histograms showing the numbers of cells and fluorescence intensity of GFP and RFP measured by flow cytometry analysis. (C) Quantification of GFP and RFP positive cells from (B). (D) To determine the impact of HA15 on the establishment of latency, uninfected iSLK cells were pre-treated with HA15 for 24h before infection with KSHV.219 at high and low MOIs. Cells were incubated for 72h, and viral genome levels were quantified by qPCR of LANA using total DNA as input. GAPDH amplification was used for normalization. N = 3. (TIF) [file ppat.1012660.s003.tif]

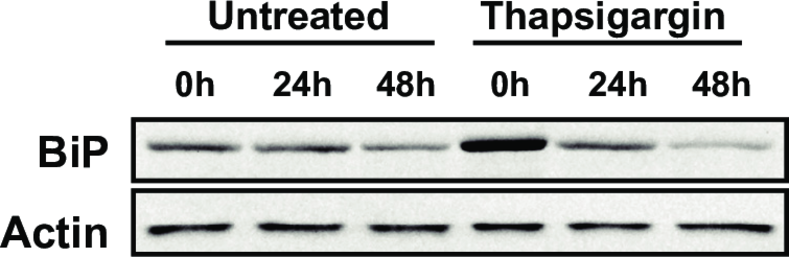

Supplement: S4 Fig — TREx-BCBL-1 cells were reactivated with Dox (2 μg/ml). At 4h before collection, cells were treated with Tg (100 nM) for 4h to induce acute ER stress. Whole-cell lysates were collected at the indicated times. Actin: loading control. (TIF) [file ppat.1012660.s004.tif]

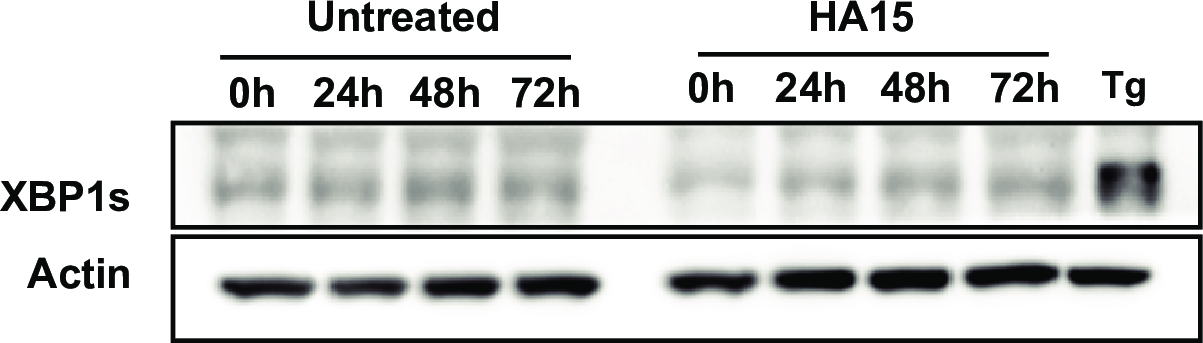

Supplement: S5 Fig — Latent iSLK.219 cells were reactivated in the presence or absence of HA15 (10μM). Whole-cell lysates collected at the indicated times were analyzed by immunoblot using an antibody specific for XBP1s. Actin: loading control. (TIF) [file ppat.1012660.s005.tif]

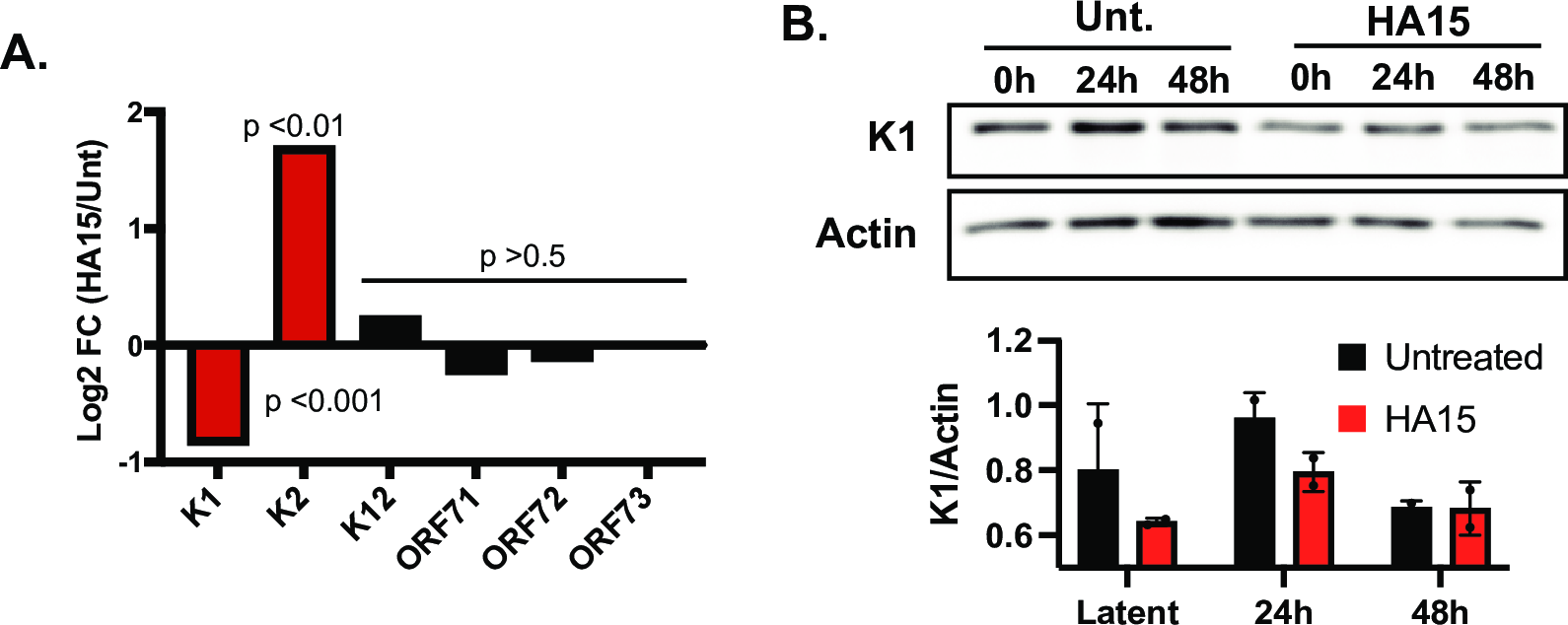

Supplement: S6 Fig — (A) Log2 fold change of latent transcripts levels in iSLK.219 cells treated with HA15 for 48h vs. untreated. (B) HA15 treatment reduces K1 levels during the KSHV lytic cycle. (top) TREx-BCBL-1-RTA cells were treated with HA15 (10 μM) 24h before induction with Dox (1 ug/ml). At 48h post-infection, whole cell lysates were collected and analyzed by immunoblot. Actin: loading control. (bottom) Image quantification by gel densitometry of the K1 immunoblot. N = 3 independent biological replicates. Values in (C) are average ±SD. (TIF) [file ppat.1012660.s006.tif]

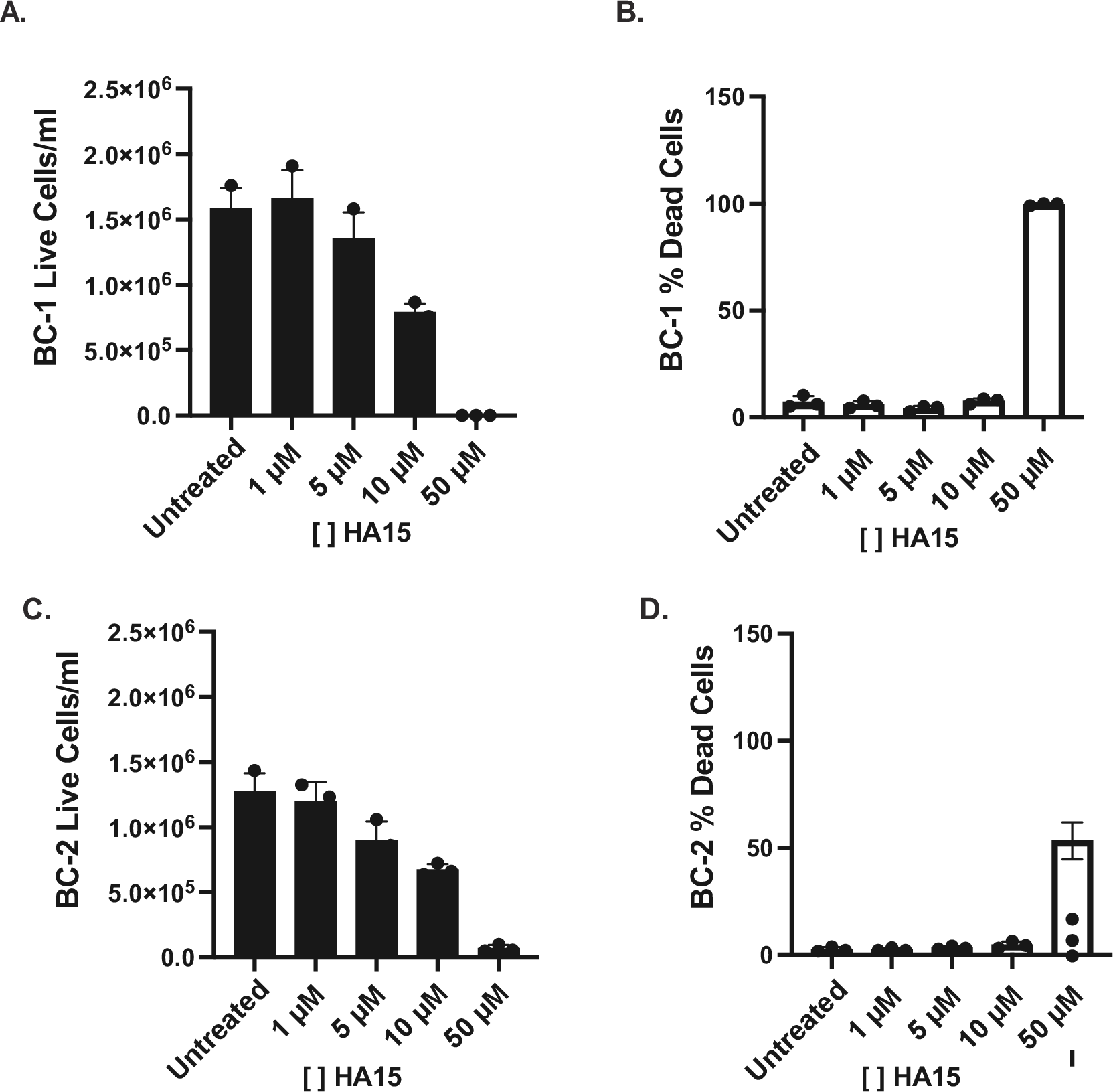

Supplement: S7 Fig — (A-D) HA15 treatment causes cytostasis in BC-1 and BC-2 cells latently co-infected with KSHV and EBV. Cells were treated with increasing doses of HA15 (0–50 μM) for 72h. The total number of viable (A) and the percent of dead BC-1 cells (B) were determined by automated cell counting following trypan blue staining. The total number of viable (C) and the percent of dead BC-2 cells (D) were determined by automated cell counting following trypan blue staining. N = 3 independent biological replicates. Values are average ±SEM. (TIF) [file ppat.1012660.s007.tif]
